# Phase, Microstructure and Corrosion Behaviour of Al_0.3_FeCoNiCr_x_ High-Entropy Alloys via Cr Addition

**DOI:** 10.3390/ma17215259

**Published:** 2024-10-29

**Authors:** Mengyao Chen, Haicheng Shen, Cheng Wen, Nanchuan Wang, Yuwan Tian, Weihua Zhong, Haiyou Huang

**Affiliations:** 1 China Institute of Atomic Energy, Beijing 102413, China; 2 College of Mechanical Engineering, Guangdong Ocean University, Zhanjiang 524000, China; 3 Zhanjiang Key Laboratory of Corrosion and Protection for Ocean Engineering and Equipment, Guangdong Ocean University, Zhanjiang 524000, China; 4 Institute for Advanced Materials and Technology, University of Science and Technology Beijing, Beijing 100083, China

**Keywords:** AlFeCoNiCr high-entropy alloy, corrosion, Cr effect, microstructure

## Abstract

The microstructure evolution of Al_0.3_FeCoNiCr_x_ (x = 0, 0.3, 0.5, 1, 1.5) high-entropy alloys (HEAs) were studied using X-Ray diffraction technique and scanning electron microscope equipped with energy dispersive spectrometer. The short-term and long-term corrosion behaviours of these alloys in 3.5 wt.% NaCl solution were studied by electrochemical impedance spectroscopy, potentiodynamic polarisation measurement, immersion test and corrosion morphology analysis. The results show that all the designed HEAs present single-phase FCC structure, and the increase in Cr content changes the microstructural morphology from cellular to a typical dendritic–interdendritic state. Without the influence of phase transformation, the corrosion resistance of Al_0.3_FeCoNiCr_x_ HEAs gradually increases with the increase in Cr content. Our designed alloys exhibit excellent corrosion resistance compared to the existing HEAs in the AlFeCoNiCr composition system.

## 1. Introduction

The design of traditional alloys typically relies on one or two primary elements, with the minor addition of other elements to regulate properties such as strength, ductility, corrosion resistance and so on, such as steel, aluminium alloys and titanium alloys. This single-primary-element design approach inevitably imposes limitations on alloy composition design. In view of this, Yeh proposed the concept of high-entropy alloys (HEAs) in 2004 [1], which were denoted as multi-principal elements alloys composed of five or more elements, with each element’s content ranging from 5 at.% to 35 at.%. HEAs concept significantly expands the compositional space for alloy design and provides new ideas for the development of novel alloy materials. Compared to traditional alloys, HEAs exhibit a range of exceptional properties, such as outstanding corrosion resistance, remarkable high-temperature strength, and good low-temperature plasticity [2,3,4,5,6]. These characteristics suggest significant potential for application in fields such as aerospace and marine structures. Developing new corrosion-resistant alloy materials is crucial for enhancing the durability of marine engineering structures and advancing the marine industry. HEAs, noted for their excellent corrosion resistance, have garnered considerable attention in recent years, with extensive studies on the oceanic corrosion behaviours of transition metal HEAs, typically as the AlFeCoNiCr composition system.

By incorporating elements such as Al, Mo, Ti and Cu, researchers have investigated the corrosion behaviour evolution of FeCoNiCr-based HEAs in various solutions containing Cl^−^, acids and bases [7,8,9]. For instance, Shi et al. [10] examined the effects of increased Al content on the microstructural evolution and corrosion performance of Al_x_CoCrFeNi HEAs, revealing that a higher Al content increases the proportion of the (Al, Ni)-rich and Cr-poor BCC phase, which reduces the alloy’s resistance to localised corrosion. The research of Zhou et al. [11] has shown that the addition of Ti promotes the formation of an oxide film, thereby reducing the corrosion current density of FeCoNiCrTi alloys in a 3.5% NaCl solution. Chou et al. [12] investigated the pitting behaviour of Co_1.5_CrFeNi_1.5_Ti_0.5_Mo_0.1_ alloys in Cl^−^ containing solutions, elucidating the mechanism by which the formation of Mo oxides enhances the alloy’s pitting resistance. Additionally, Liu et al. [13] found that a moderate increase in Mo content in Al_x_CrFeNi_2.5_Mo_1−x_ HEAs inhibits the formation of the (Al, Ni)-rich and Cr-poor BCC phase, thereby improving the alloy’s corrosion resistance. However, Linder et al. [8] revealed that increasing the Mo content in (FeCoNiCr)_1−x_Mo_x_ alloys resulted in a detrimental effect on the alloy’s corrosion performance due to an increase in the proportion of the σ phase. Most research shows that the incorporation of different elements impacts the phase structure and microstructure evolution of HEAs with various composition systems, leading to differences in corrosion behaviour. In fact, the effect of the same element on the corrosion resistance of different HEA systems often varies. The positive effect of some elements on corrosion resistance in a certain composition system may be negative in another system, typically, such as Mo for HEAs in FeNiCrAl-base [13], FeNiCrCo-base [12] and FeNiCrCoTi-base [8] composition system.

As a typical passivation-promoting element for traditional corrosion-resistant steel design, Cr addition is usually considered to be favourable to corrosion resistance improvement of HEAs. However, Cr is also prone to inducing compositional segregation and the precipitation of Cr-rich phases, which can adversely affect the localised corrosion resistance of HEAs. From the investigation of Chai et al. [14], the addition of excessive Cr led to element segregation and an uneven distribution of Cr-rich and Cr-poor regions, ultimately resulting in a decrease in the corrosion resistance of FeCoNiCr_x_ HEAs. Tsau et al. [15] reported that the corrosion resistance of Cr-modified FeCoNi alloys, specifically FeCoNiCr with high Cr content, was inferior to that of FeCoNi without Cr in both 1 M H_2_SO_4_ and NaCl solutions. The addition of Cr led to the formation of hexagonal close-packed (HCP) precipitates within the alloy matrix, contributing to the deterioration of corrosion performance. The study by Jiang et al. [16] indicated that the effect of Cr addition on the corrosion performance of AlFeCoNiCr_x_ HEAs coatings exhibited a nonlinear relationship. According to the results of electrochemical tests and immersion tests, the corrosion resistance of the alloy coatings in 3.5 wt.% NaCl solutions initially increase with the increasing Cr content, followed by a subsequent decline. The authors asserted that the enhanced corrosion resistance of AlFeCoNiCr_x_ coatings was a result of the combined effects of Cr and Al elements. The increase in Cr content within the passivation film cannot compensate for the decrease in corrosion resistance caused by the reduction of Al content. Consequently, the coating with the highest Cr content demonstrated poorer corrosion resistance. Qiu et al. [17] also pointed out that Cr segregation can lead to the precipitation of the Cr-rich σ phase, which makes the Cr-depleted HEAs matrix more prone to corrosion. Additionally, due to the interaction between multiple elements in HEAs for different composition systems, the effect of the Cr element on improving corrosion resistance is still vague and needs to be investigated. Actually, phase transformation in HEAs caused by Cr addition can influence the corrosion performance of HEAs and may lead to galvanic corrosion between the solid solution matrix and the precipitation phase [18]. The corrosion behaviour of Cr-containing HEAs exhibits unique characteristics.

The development of homogeneous solid solution alloys that minimise structural heterogeneities represents the most effective strategy for designing corrosion-resistant alloys (CRAs) with the highest potential for superior corrosion resistance [19]. One significant approach in the design of CRAs has been the development of homogeneous solid solution alloys that emphasise phase and compositional stability while minimising structural non-uniformities.

Previous studies on Cr-containing HEAs did not pay attention to this point, resulting in excessive Cr content addition that actually reduces the corrosion resistance. The goal of this study is to gain a deeper understanding of the corrosion behaviour of HEAs as a function of chemical composition (specifically Cr itself) while maintaining a fixed single-phase structure. We intend to eliminate the influence of phase transformation caused by increased Cr content on corrosion behaviour and explore the enhancing effect of the Cr element itself on corrosion performance; thus, a series of single-phase HEAs, denoted as Al_0.3_FeCoNiCr_x_, with varying Cr contents were designed based on empirical rules. The phase structure and microstructural characteristics of the alloys were analysed using techniques such as X-Ray diffraction (XRD) and scanning electron microscopy (SEM). Furthermore, the effects of increased Cr content on the short-term and long-term corrosion behaviour of the alloys were investigated through potentiodynamic polarization measurements, impedance analysis and immersion tests. The positive effect of Cr addition on the corrosion performance of HEAs with single-phase solid solution is demonstrated. In comparison to the reported HEAs within the AlFeCoNiCr compositional system, our designed single-phase alloys show outstanding corrosion resistance in marine environments.

## 2. Materials and Methods

### 2.1. HEAs Preparation

The Al_0.3_FeCoNiCr_x_ (x = 0, 0.3, 0.5, 1 and 1.5) HEAs were prepared by arc melting in a Ti-gettered argon atmosphere, utilizing high-purity Al, Fe, Co, Ni and Cr elements (purity ≥ 99.9 wt.%). The melting current is around 200A, with a slight difference for the five alloys. During each melting process, the alloys were maintained in a liquid state for 3 min, simultaneously conducting electromagnetic stirring to accelerate liquid flow. The ingot was then rapidly solidified in a copper mould. The alloys were melted five times, and the ingot was flipped after each melting process to ensure chemical homogeneity. Square pieces measuring 10 mm × 10 mm × 5 mm were cut from the bulk ingot for phase structure and microstructural analysis. For simplification, the five alloys were designated as Cr0 (x = 0), Cr3 (x = 0.3), Cr5 (x = 0.5), Cr10 (x = 1) and Cr15 (x = 1.5), respectively.

### 2.2. Characterization of Phase and Microstructure

The phase structures of the Al_0.3_FeCoNiCr_x_ HEAs were detected with an X-Ray diffraction technique (XRD) using Bruker D8 Advancey equipment (Karlsruhe, Germany), and the detailed parameters were as follows: Cu Kα radiation, scanning angle 20~100° and scanning rate 6°/min. A scanning electron microscope (SEM) equipped with EDS was employed to characterise the samples’ microstructure, pitting corrosion morphology, and element distribution. The specimens for microstructure observation were ground using silicon carbide abrasive papers from grit 240 to 2000 and polished with 1.5 μm diamond polishing agents, then etched in a mixture of nitric acid and hydrofluoric acid aqueous solution with a volume ratio of 1:1:2.

### 2.3. Electrochemical Measurement and Immersion Test

The specimens for the electrochemical measurements were cut into 10 mm × 10 mm × 5 mm bulks and cold-mounted in epoxy resin with copper wires spot-welded to the back for electrical contact. Prior to each electrochemical experiment, the exposed surface of the specimens was wet ground with a series of SiC papers ranging from 240 to 2000 grit, thoroughly cleaned with alcohol, acetone and deionised water in sequence. Both electrochemical polarisation and electrochemical impedance spectroscopy (EIS) were conducted using an Autolab 302N electrochemical workstation in a three-electrode system (Herisau, Switzerland). The specimen served as the working electrode, a saturated calomel reference electrode (SCE) was used as the reference electrode, and a platinum counter electrode was used. During the experiment, the working electrode was initially cathodically polarised at −800 mV vs. SCE for 5 min to reduce any possible surface oxides. The sample was then allowed to corrode freely for 30 min to reach a quasi-stationary open circuit potential (OCP). The potentiodynamic-polarisation tests were performed at scanning rates varying from 10 mV/min, starting from an initial potential of −250 mV vs. OCP until the current density reached a maximum of 1 mA/cm^2^. The EIS tests were carried out at the OCP with a sinusoidal potential amplitude of 10 mV, operating from 100 kHz to 10 mHz.

Additionally, to further investigate the long-term corrosion behaviour of the designed HEAs, the electrochemical samples were immersed in a 3.5 wt.% NaCl solution at room temperature for 0, 1, 3, 5 and 7 days. Subsequently, the impedance was tested at each interval. The surface morphologies of the alloys after the immersion tests were also analysed using SEM.

## 3. Results and Discussion

### 3.1. Phase Structure

**Figure 1:**
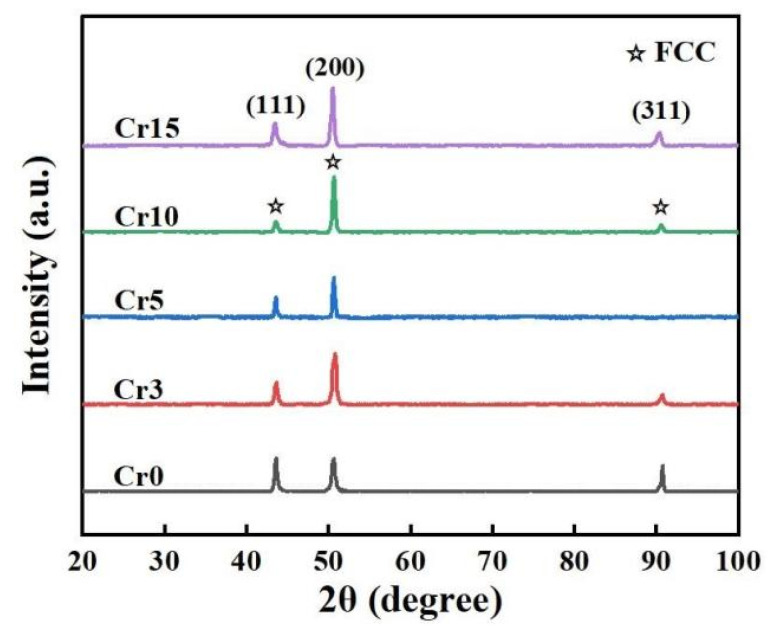
XRD patterns of the studied HEAs.

The XRD patterns presented in Figure 1 indicate that all the alloys exhibit a simple FCC solid-solution structure, characterised by three distinctive peaks corresponding to the (1, 1, 1), (2, 0, 0) and (3, 1, 1) crystal planes, as anticipated. Exploring the phase formation rules and predicting the phase structure has long been a pivotal topic in the realm of HEAs. To address this, researchers have proposed numerous empirical parameters for phase prediction in HEAs, primarily derived from Hume-Rothery rules and thermodynamic criteria. These criteria are determined by the physicochemical and thermodynamic properties of the constituent alloying elements in HEAs, with the parameter ranges for phase selection often being empirical values based on diverse datasets.

The calculated parameters for the studied HEAs are detailed in Table 1, from which it is evident that all five alloys meet the thermodynamic parameter criteria for solid solution formation in HEAs, specifically −22 < ΔH_mix_ < 7 and 11 < ΔS_mix_ < 19.5, as referenced in [20]. It is widely acknowledged that δr (atomic size mismatch) serves as a valuable empirical parameter for phase prediction in HEAs. However, varying research groups have occasionally proposed different threshold values. Ren et al. [21] asserted that solid solutions were formed when δr < 0.028 and ΔH_mix_ > −8.8, significantly narrowing the δr-ΔH_mix_ space compared to Yang’s research (δr < 0.066) [22]. This divergence arises because Yang’s work classifies ordered solid solutions as solid solutions rather than intermetallic compounds. Kube’s research [23] highlights that HEAs with increasing δr values tend to favour a BCC structure over FCC, attributed to the BCC structure’s capacity to accommodate larger atomic size differences with lower strain energy. As seen in Table 1, the studied alloys exhibit low δr values, signifying a high tendency for the formation of stable FCC solid solutions, which aligns with the XRD results.

Valence electron concentration (VEC) has been proposed as a crucial parameter to control the phase stability of HEAs, distinguishing between FCC and BCC solid solutions [24]. The stability of the FCC phase is observed at higher VEC (>8), whereas the BCC phase is stable at lower VEC (<6.87). By examining the relationship between electronic concentration and solid solution phase formation in HEAs, Battezzati et al. [25] provided a new guideline for selecting the set of elements to obtain different solid solution phases of HEAs based on data analysis using a broader range of alloy samples. In this research, the VEC value requirement for HEAs forming a single-phase FCC structure is greater than 7.5, and all the studied alloys meet this threshold. However, atoms with different electron affinities tend to form ordered intermetallics according to the Hume-Rothery rules. Guo’s research [20] points out that Δχ (Pauling electronegativity difference) seems to have no significant influence on solid solution phase formation in HEAs. However, the newly proposed Δχ_Allen_ (Allen electronegativity difference) indicates that for HEAs with 0.001 < δr < 0.006 and 3% < Δχ_Allen_ < 6%, only solid solutions are formed [25]. The XRD results are consistent with this parameter limit, reinforcing the importance of Δχ_Allen_ in predicting the phase stability of HEAs.

Similar parameters like Ω [22], Φ [26], Λ [27], s_m_ and K_m_ [28] were also proposed from the aspects of thermodynamics, lattice distortion and electronic behaviour of components to try to explain the phase formation of HEAs. For the studied HEAs, Cr is an element that stabilises the BCC solid solution phase [29]. As the content of Cr increases, δr continuously decreases, which enhances the tendency of the alloy to form an FCC solid solution. Conversely, the continual decrease in VEC reduces the inclination of the alloy to form an FCC solid solution. Additionally, the increasing Δχ_Allen_ raises the propensity for the formation of intermetallic compounds in the alloy. In sum, phase prediction and design of HEAs is a complex issue that has yet to be solved. Even though there are no parameters that can accurately determine the HEAs phases up to now, the parametric approach is still considered a good reference for rapid composition design in HEAs.

### 3.2. Microstructure

**Figure 2:**
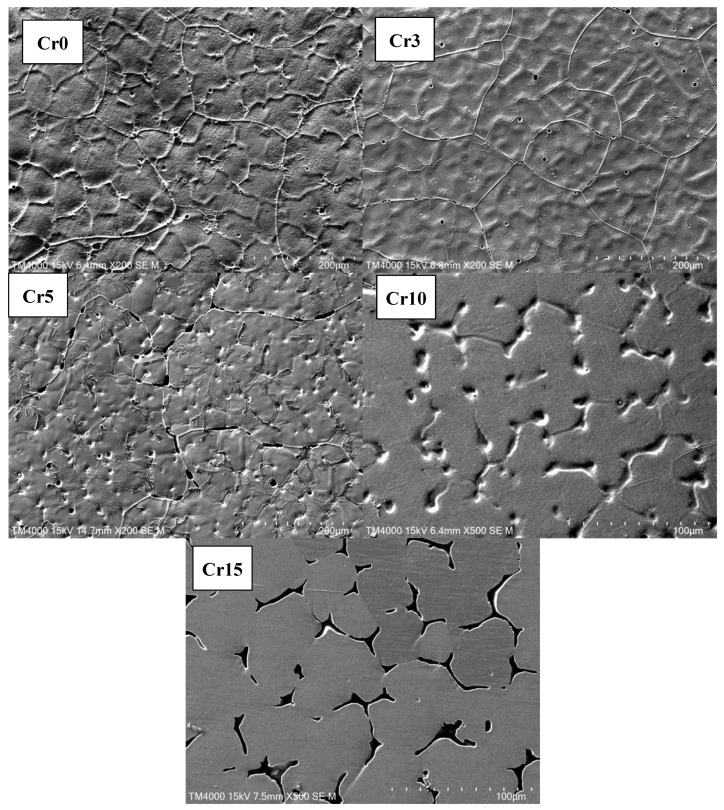
SEM micrographs of the studied HEAs.

**Figure 3:**
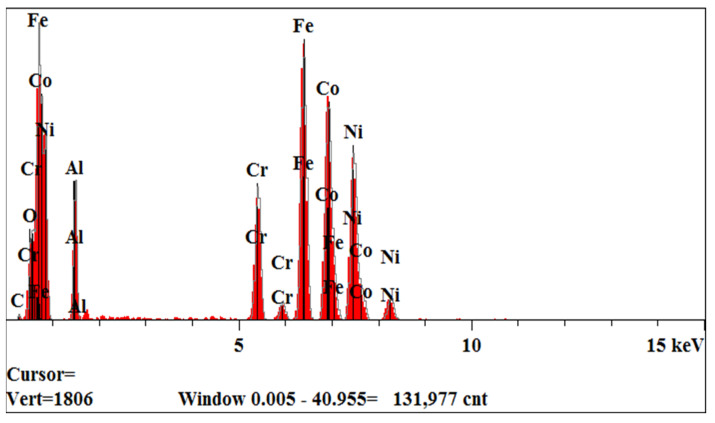
Element detection of the studied HEAs using the EDS technique.

The cross-sections perpendicular to the direction of heat flow of the as-cast samples were used for the microstructure observation. Figure 2 displays the SEM micrographs of Al_0.3_FeCoNiCr_x_ alloys in secondary electronic mode. It can be seen that Cr0, Cr3 and Cr5 present a uniform and fine cellular structure. With the increase in Cr content, the alloys Cr10 and Cr15 exhibit a typical dendritic–interdendritic state. The increase in Cr content changes the microstructural morphology from cellular to dendrite, similar to the previous research on microstructure evolution for Al-free FeCoNiCr_x_ HEAs [14]. From the viewpoint of solidification, the cellular-to-dendrite transition is mainly attributed to the instability of the solid/liquid interface caused by element microsegregation, which may be detrimental to the localised corrosion resistance of alloys. We will discuss it in the following part. As shown in Figure 3, we utilised the EDS technique for the analysis of the alloy composition, with a comparison of the detected elemental concentrations and the nominal compositions presented in Table 2. It can be observed that the average composition of the prepared alloy is close to the designed composition, with deviations in elemental concentrations generally within 5%. Furthermore, the elemental analysis indicates that with the increase in Cr content, there is a tendency for greater compositional segregation in the alloy, although the degree of segregation remains minimal. It can be inferred that, for the alloys in this study, the effects of compositional segregation and phase structure changes due to the increase in Cr on the corrosion performance are relatively small. Thus, we can explore the influence of Cr itself on the alloy’s corrosion behaviour and corrosion resistance.

### 3.3. Short-Term Corrosion Behaviour

**Figure 4:**
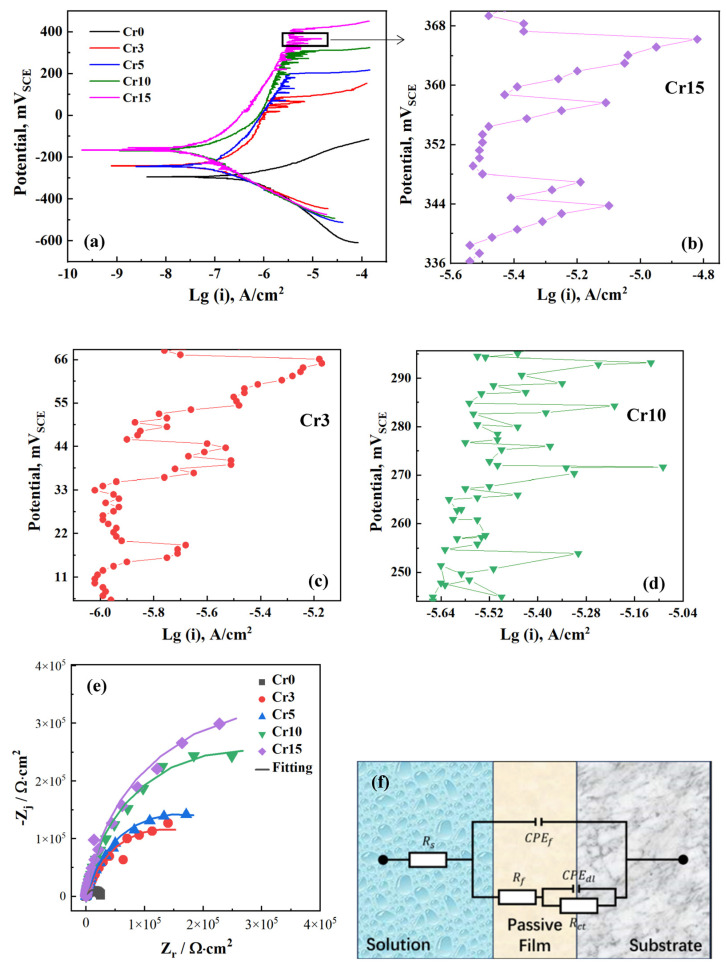
Electrochemical test result: (**a**) Potentiodynamic polarisation curves; (**b**) Localised magnification of polarisation test within the black box for Cr15 (**b**), Cr3 (**c**) and Cr10 (**d**); (**e**) Nyquist plots; (**f**) equivalent electron circuit model of the EIS data fitting.

Figure 4a displays the typical potentiodynamic polarisation curves of the Al_0.3_FeCoNiCr_x_ HEAs in 3.5 wt.% NaCl. The corrosion current densities of the alloys, determined using the Tafel extrapolation method, are presented in Table 3. From Figure 4a, it is evident that the designed alloy Cr0 without Cr exhibits pronounced active corrosion behaviour. The anodic current density gradually increases with the potential, showing no signs of depassivation or pitting corrosion. In contrast, all the Cr-containing alloys exhibited passivity and breakdown behaviour, as indicated by a slowly increasing anodic current density followed by a sudden surge in current when the potential reached a certain value. It can be seen that all four alloys have obvious passivation zones, and the polarisation curves directly transition from the Tafel zone to the passivation zone without any activation passivation transition process, indicating the natural formation of a protective passivation film under open circuit potential. In the passivation zone, the passivation current density of the four alloys is relatively low, with an order of magnitude of 10^−6^ A/cm^2^.

The Cr element combines a relatively high heat of adsorption for oxygen (or hydroxide) with a comparatively low Cr-Cr bond strength [30], thus the enhancement of passivation in alloys can be achieved through Cr addition. Although Al belongs to this type of element, a passive state is absent in the curves obtained for the Cr0 alloy with 9 at.% Al content due to the lack of Cr. Even with higher Al content, there is no evidence of passivation behaviour in the AlCoCr_0.5_FeNi alloy coating with low Cr content [16]. Actually, compared to Al, Cr plays a decisive role in improving corrosion resistance in such HEA composition systems through the formation of a steady-state passivation film. For the studied HEAs, higher Cr content indicates higher breakdown potential. According to Table 3, the larger passivation interval further demonstrates the positive effect of Cr addition on corrosion protection for the single-phase HEAs. It is generally considered that the Cr content should be more than 12 at.% to ensure passivation for traditional Fe-Cr-Ni alloys. From the previous research of Lu et al. [31], HEAs containing 6 at.% Cr showed signs of passivity, which was significantly lower than the Cr threshold passivation level. Our work also demonstrates that from Cr0 to Cr3, an increase of 8.3 at.% in Cr content can achieve the passivation protection of alloys.

From Figure 4a, for all the Cr-containing HEAs, at potentials of 100 to 200 mV below their pitting potentials, numerous current transients are evident in the passive region, indicative of metastable pitting [31,32,33]. The occurrence of metastable pitting can be attributed to the fact that, along with the increase in applied potential, the insoluble oxides in the passive film may be transformed into soluble chlorides due to O in the oxides being replaced by Cl^−^ in the NaCl solution. This transformation leads to the initiation of pitting and an increase in anodic current density. However, under the strong passivation effect of the Cr element, the pitting is repaired, resulting in a subsequent decline in anodic current density. A further increase in the anodic overpotential progressively raises both the magnitude and number of the metastable pits until the current suddenly increases. Typically, Figure 4b–d depict the localised magnification of the polarisation curve for the Cr15, Cr3 and Cr10 alloys, which belongs to a typical class of current density fluctuation behaviour observed in metastable pitting corrosion for traditional CRAs [32]. The current density slowly increases due to the anodic dissolution of the metal inside the pitting corrosion during the development of metastable pitting corrosion. Once re-passivation occurs after a period of time due to metal dissolution, the current density sharply decreases. In fact, the higher the Cr content, the faster the response to re-passivation repair is. As the Cr content increases, the current fluctuation from metal dissolution to re-passivation decreases, indicating the improved stability of the protective passivation film. The stability of re-passivation repair brought by the increase in Cr content is very important for the long-term corrosion of the investigated HEAs. Li et al. [34] also observed similar electrochemical behaviours in the polarisation measurements. The frequent breakdown and re-passivation events in the passive region suggest that the passive films formed on these alloys are susceptible to Cl^−^-induced instabilities. This susceptibility is likely associated with structural defects in the as-cast condition, such as shrinkage porosity and shrinkage cavities, rather than element segregation. A similar phenomenon also occurs in HEAs like CoCrFeNi and CoCrFeNiMn, which possess uniform compositions after deformation and heat treatment [35].

In terms of corrosion current density, it is evident that Cr significantly enhances the corrosion resistance of HEAs, and this improvement increases with rising Cr content, which is similar to the behaviour observed in Cr-containing steel. The corrosion current densities for Cr3, Cr5, and Cr10 decrease by approximately 5.5 times compared to that of Cr0, while the corrosion current density for Cr15 decreases by about 11 times relative to Cr0. It can be seen that the addition of merely 8.3 at.% Cr (Cr3) to the Al_0.3_FeCoNiCr_x_ HEAs can achieve a notable enhancement in corrosion resistance. However, the Cr content was further increased to 23.3 at.% (Cr10) does not yield significant changes in corrosion resistance. When the Cr content is raised to 31.3.% (Cr15), the corrosion resistance of the alloy improves once more. From the above analysis, although the corrosion resistance of the designed alloys continues to improve with the increased Cr, the degree of improvement weakens. The corrosion performance indicators corresponding to the unit Cr addition are decreasing, especially when the cellular-dendritic transition occurs (Cr5 to Cr10). The most obvious change in pitting potential (E_pit_) and passivation interval (E_pit_-E_corr_) is observed, indicating that the excess Cr addition may lead to localised corrosion due to the formation of dendritic microstructure. From Figure 4c,d, compared to the Cr3 alloy with a cellular microstructure, the Cr10 alloy exhibits more severe current fluctuations during the metastable pitting stage. The cellular-dendritic transformation caused by excess Cr addition is unfavourable to localised corrosion, which may not be compensated by the passivation-promoting effect of the Cr element itself.

Figure 4e depicts the EIS results of the studied alloys. From the Nyquist plot, all impedances exhibit similar capacitive arcs, indicating that the alloys possess comparable interfacial electrical properties and no mass transportation processes are observed [36,37,38]. It is generally accepted that a larger radius of the capacitive arc corresponds to better corrosion resistance of the alloys [36,39,40]. Therefore, the increase in the radius of the capacitive arc with rising Cr content also suggests that Cr addition effectively enhances the corrosion resistance of the present HEAs. Furthermore, the EIS data were fitted using the circuit shown in Figure 4f. Due to the passivation behaviour observed in the polarisation curves and the broad phase angle peak evident in the Bode plot, a fitting circuit with two time constants was employed. The peak in the phase angle-frequency plot in the 1~10 Hz range corresponds to the charge transfer mechanism and the double layer capacitance effect at the metal surface, while the notable dispersion phenomenon or recognisable time constant at around 100 Hz correlates with the properties of the surface film on the metal. Consequently, in Figure 4f, Rs represents the solution resistance, while CPEf and Rf denote the film capacitance and film resistance of the metal surface, respectively. CPEdl and Rct represent the double-layer capacitance and charge transfer resistance at the metal surface.

The EIS data-fitting results are shown in Table 4; it also presents the polarisation resistance (Rp = Rf + Rct) to characterise the corrosion resistance of the passivated samples [41,42,43]. It is evident that the values of Rct are 1~3 orders of magnitude higher than Rf, indicating that the charge transfer process is a significant factor controlling the overall corrosion kinetics in this system. The Rct values for Cr3, Cr5, Cr10 and Cr15 are approximately 14, 16, 28 and 36 times higher than that of Cr0, respectively, and the Rp values are about 10, 12, 21 and 26 times higher, respectively. The impedance analysis also indicates that there is an addition of merely 8.3 at.% Cr (Cr3) can significantly enhance the corrosion resistance of the present HEAs. Further increasing the Cr content to 13.2 at.% (Cr5) results in a marginal change in corrosion resistance while increasing the Cr content to 23.3 at.% (Cr10) improves the corrosion resistance again. This conclusion is consistent with the results from the polarisation curves. Overall, the results from polarisation curves and EIS corroborate each other, fully validating the effectiveness of Cr in reducing the corrosion rate of HEAs. Moreover, this enhancing effect persists and even strengthens with increasing Cr content for the present single-phase alloys.

### 3.4. Long-Term Corrosion Behaviour

**Figure 5:**
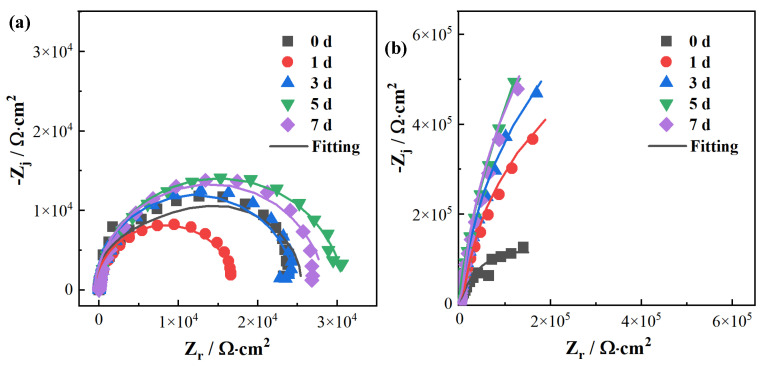

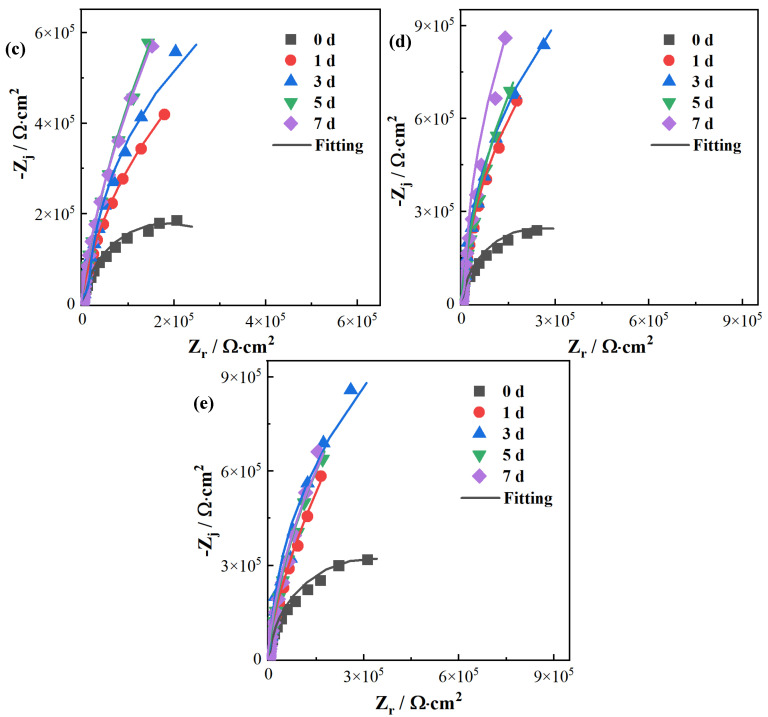
Nyquist plot of the five alloys immersed in 3.5 wt.% NaCl solution for 0, 1, 3, 5 and 7 d: (**a**) Cr0, (**b**) Cr3, (**c**) Cr5, (**d**) Cr10, (**e**) Cr15.

To further investigate the long-term corrosion behaviour of the designed HEAs, the electrochemical samples were immersed in a 3.5 wt.% NaCl solution at room temperature for seven days. Non-destructive EIS tests were conducted for the five alloy samples on 0, 1, 3, 5, and 7 d, with results displayed in Figure 5. It can be observed that the impedance value and the radius of the capacitive arc for the Cr0 alloy decrease on the first day, indicating that corrosion occurs on its surface. After day 3, the impedance value and the radius of the capacitive arc increase, which may be associated with a certain amount of corrosion products covering the sample surface. From day 3 onwards, the changes in impedance value and radius of the capacitive arc for Cr0 are minimal, and the low-frequency impedance stabilise at 25 kΩ·cm^2^, suggesting that it experiences continuous corrosion at a relatively stable rate.

In comparison, Cr3 and Cr5 exhibit significant differences from Cr0. Firstly, their impedance values and capacitive arc radii markedly increase on day 1, indicating that the addition of a small amount of Cr effectively promotes passivation of the metal surface in a Cl^−^-containing solution environment. During days 3 to 7, the impedance values and capacitive arc radii continue to increase slowly, suggesting that the passivation film on the metal surface thickens, becomes denser and increases in protective quality. Towards the later stages of immersion, the low-frequency impedance values for Cr3 and Cr5 notably increase to 200 kΩ·cm^2^, which represents an order of magnitude improvement compared to the 25 kΩ·cm^2^ of Cr0, thus confirming that a small amount of Cr significantly enhances the corrosion resistance of the studied HEAs. For Cr10 and Cr15, the evolution of their impedance spectra was similar to that of Cr3 and Cr5, with slight differences: the impedance spectra for Cr10 and Cr15 reached elevated values on the first day of immersion and remained relatively stable during the immersion period from days 3 to 7. This indicates that an increase in Cr content can accelerate the rate of passivation on the metal surface.

Similarly, the EIS data of the alloy samples were fitted using the equivalent circuit with two time constants shown in Figure 4c, and the results are presented in Table 5. It can be seen that the variation pattern of the polarisation resistance for Cr0, Cr3, Cr5, Cr10 and Cr15 is consistent with that of the low-frequency impedance values: all of them show a slowly increasing trend over time, indicating that their surface corrosion rates are gradually decreasing. The polarisation resistance of Cr0 remains in the order of 10 kΩ·cm^2^, while the polarisation resistance of the HEAs with Cr addition is in the order of 1 MΩ·cm^2^, and may even reach 10 MΩ·cm^2^ in the later stages, further confirming the continuous role of the alloying element Cr in enhancing the corrosion resistance of the studied HEAs.

**Figure 6:**
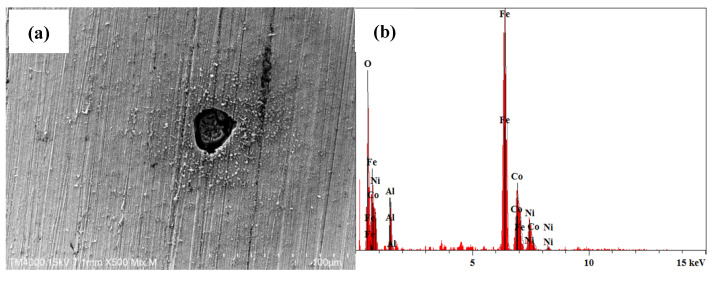

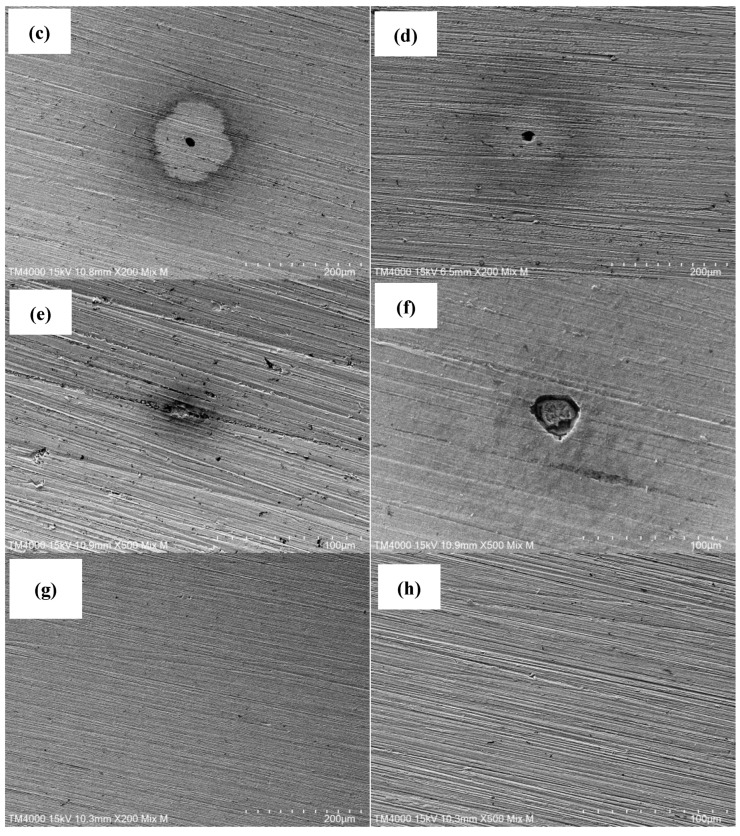
SEM micrographs of the alloy surface after immersion test: (**a**) Cr0 immersion for 1 d and its EDS analysis (**b**); (**c**–**f**) Cr0 immersion for 7 d; (**g**) Cr3 immersion for 7 d; (**h**) Cr10 immersion for 7 d.

Furthermore, the corrosion morphologies of the alloys after immersion experiments were observed using SEM, and the results are shown in Figure 6. From Figure 6a, after 1 day of immersion, the Cr0 sample displays a distinct pitting pit, and EDS analysis indicates that it is primarily composed of Fe oxides. As the immersion time increased, no new pitting pits were observed until the 7th day of immersion, at which various pit morphologies emerged, as depicted in Figure 6c–f. This result is consistent with the impedance tests conducted after the immersion experiment for Cr0, where impedance decreases after 1 day of immersion, followed by a gradual stabilisation of the surface and an increase in impedance with prolonged immersion time. New pits appeared after the 7th day, leading to a subsequent decrease in impedance. Regarding the Cr-containing HEAs, no pitting phenomenon was observed during the immersion test, as shown in Figure 6g,h. For Cr3 to Cr15, a protective film gradually formed on the alloys’ surface with increasing immersion time, as evidenced by the continuous rise in impedance. However, with the increasing Cr content, the impact of immersion time on the samples’ impedance diminishes. High Cr content can foster the formation of the protective film on alloy surfaces more rapidly.

### 3.5. Corrosion Property Comparison

**Figure 7:**
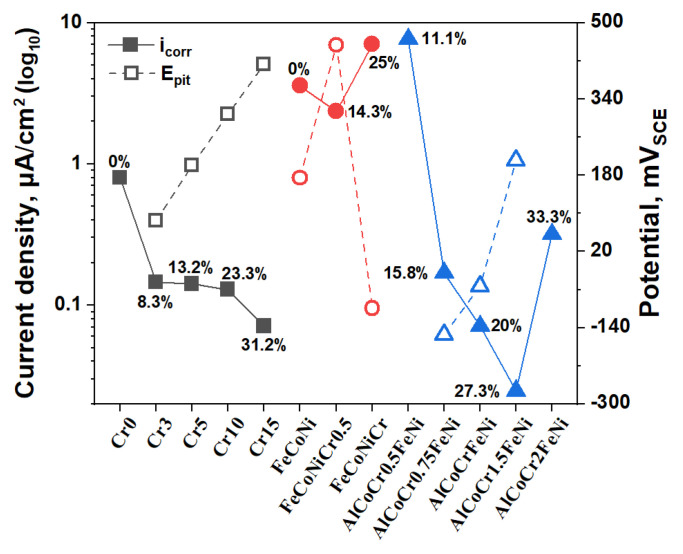
The corrosion properties comparison between the studied alloys and the reported Cr-containing HEAs. The square symbol represents the studied alloys system. The circle symbol represents the FeCoNiCr_x_ alloys system. The triangle symbol represents the AlCoCr_x_FeNi alloys system.

Figure 7 illustrates the comparison of corrosion current density and pitting potential in a 3.5 wt.% NaCl solution between the studied alloys and the reported Cr-containing HEAs, demonstrating the effect of the Cr element on corrosion properties. It is evident that with increasing Cr content, the pitting potential of the alloys continuously rises while the corrosion current density generally declines. A minimal addition of Cr (8.3 at.%) significantly enhances the corrosion resistance of the alloys, which remains relatively stable from Cr3 to Cr10. This indicates that under single-phase conditions, the addition of Cr exerts a positive effect on improving the corrosion resistance of HEAs. As for FeCoNiCr_x_ (x = 0, 0.5, 1) HEAs, an increase in Cr content initially results in a decrease in corrosion current density, followed by an increase. The pitting potential shows an opposite trend, with excessive Cr addition leading to localised corrosion in FeCoNiCr, thereby deteriorating corrosion resistance [15]. For AlCoFeNiCr_x_ (x = 0.5, 0.75, 1, 1.5, 2) HEAs, a minimal addition of Cr (11.1 at.%) cannot cause alloy passivation. As Cr content further increases to 15.8 at.%, the corrosion current density significantly decreases. Continuing to increase Cr content to 27.3 at.% reduce the corrosion current density, and the pitting potential continues to rise. However, further increasing Cr content leads to an increase in corrosion current density with no passivation phenomenon. This may be related to changes in the alloy phase structure and alterations in the Al/Cr ratio affecting the formation of the passivation film, resulting in decreased corrosion resistance [17]. These findings indicate that the influence of the Cr element on the corrosion resistance of HEAs correlates with their effects on phase structure changes and interactions with other elements. Only under single-phase conditions does increasing Cr content have a continuous positive effect on improving alloy corrosion performance.

**Figure 8:**
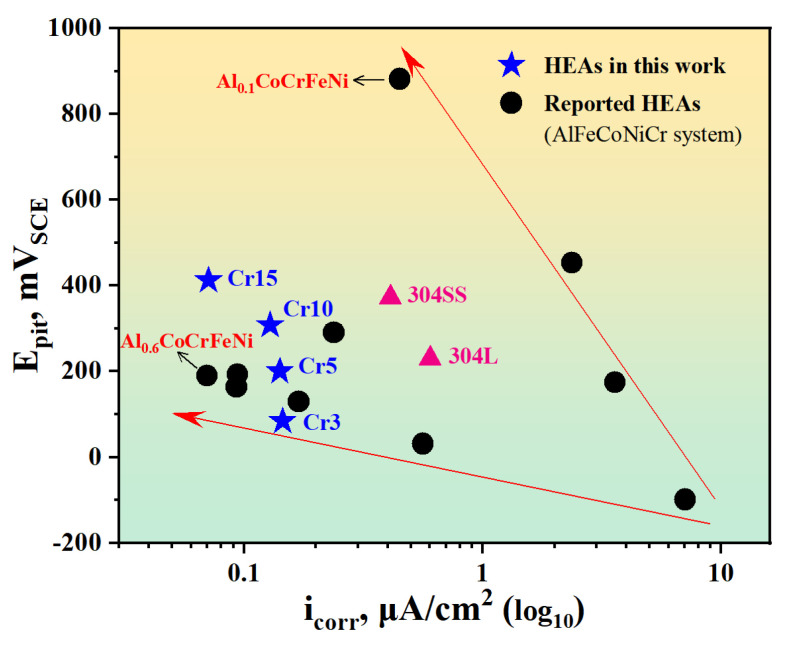
The corrosion properties comparison between the studied alloys and the reported HEAs in the AlFeCoNiCr composition system [14,15,16,44,45,46].

Furthermore, we compare the corrosion properties of our alloys with those of the reported HEAs in the AlFeCoNiCr composition system and the typical stainless steel. From Figure 8, it is evident that the designed alloys exhibit superior corrosion resistance, with lower i_corr_ and higher E_pit_ in a Cl^−^-containing environment. By adjusting the content of the Al element, the reported HEAs show low corrosion current density (Al_0.6_FeCoNiCr) or high pitting potential (Al_0.1_FeCoNiCr). In contrast, the alloys prepared in this study not only possess a lower corrosion current density, indicative of their superior resistance to general corrosion but also exhibit a higher pitting potential, signifying their notable resistance to localised corrosion in Cl^−^-containing solution.

Additionally, our developed HEAs show superior corrosion resistance even compared to the two commercial stainless steels of 304 and 304L. The Cr element content of the latter alloys is generally between 18–20 at.%, exceeding the maximum Cr content of the designed alloy. The Cr5 alloys with minor Cr exhibit lower corrosion current density and higher pitting potential than the 304L, indicating its better general corrosion and localised corrosion resistance. It can be reasonably inferred that the corrosion resistance of the investigated HEAs can be further improved after rational plastic deformation and heat treatment to eliminate possible segregation and optimise the microstructure of the as-cast alloys. It is recommended in the future work. However, the phase-formation empirical rule helps to determine the single-phase HEAs, and with the increase in Cr content, the corrosion properties of the designed alloys are continuously enhanced to reach the desired potential and current density direction. It also suggests that by judiciously regulating the composition, maybe the ratio of Cr and Al elements, it is possible to achieve higher corrosion resistance, thereby promoting the potential application of HEAs in marine environments.

## 4. Conclusions

This study systematically investigates the effect of Cr addition on the phase structure, microstructure, and corrosion performance of the Al_0.3_FeCoNiCr_x_ HEAs. The results indicate that the alloys within the designed Cr content range maintain a single-phase FCC structure consistent with most empirical criteria. With the increase in Cr content, the as-cast microstructure of the alloy transitions from cellular structures to typical dendritic–interdendritic morphologies. The results of the electrochemical and immersion test demonstrate that increasing the Cr content has a positive effect on improving the pitting potential of the alloys, reducing the corrosion current density, and enhancing the protective efficacy of the passivation film. The study shows that under single-phase conditions, the Cr element is effective in slowing down the corrosion rate of the Al_0.3_FeCoNiCr_x_ HEAs, and this improvement effect continues to strengthen with increasing Cr content, promoting the earlier formation of a protective film on the alloy surface. The designed alloys exhibit significantly superior corrosion performance compared to the current reported HEAs in the AlCoFeNiCr composition system and the commercial stainless steel of 304. In the future, by integrating empirical rules for HEAs phase formation and further synergistic regulation of Al and Cr elements, it is possible to develop HEAs with even better corrosion resistance.

## Figures and Tables

**Table 1:** Empirical parameters calculated for the studied HEAs: ΔH_mix_—mixing enthalpy, ΔS_mix_—mixing entropy, δr—atomic size mismatch, VEC—valence electron concentration, and Δχ_Allen_—Allen electronegativity difference.

Alloys	ΔH_mix_	ΔS_mix_	δr	VEC	Δχ_Allen_
Al_0.3_FeCoNi (Cr0)	−6.83	10.84	0.0425	8.45	3.97%
Al_0.3_FeCoNiCr_0.3_ (Cr3)	−7.13	12.32	0.0408	8.25	4.63%
Al_0.3_FeCoNiCr_0.5_ (Cr5)	−7.23	12.65	0.0398	8.13	4.92%
Al_0.3_FeCoNiCr (Cr10)	−7.27	12.83	0.0376	7.88	5.37%
Al_0.3_FeCoNiCr_1.5_ (Cr15)	−7.14	12.61	0.0357	7.69	5.95%

**Table 2:** EDS analysis result of the studied alloys.

	Element	Content, at.%	Al	Fe	Co	Ni	Cr
Alloys							
Cr0		Nominal	9.1	30.3	30.3	30.3	0
		EDS	8.96 ± 0.11	29.65 ± 0.27	30.12 ± 0.21	31.27 ± 0.19	0
Cr3		Nominal	8.33	27.78	27.78	27.78	8.33
		EDS	7.57 ± 0.12	27.15 ± 0.28	27.90 ± 0.18	29.18 ± 0.20	8.20 ± 0.06
Cr5		Nominal	7.88	26.32	26.32	26.32	13.16
		EDS	7.69 ± 0.13	26.95 ± 0.27	25.87 ± 0.16	25.62 ± 0.17	13.87 ± 0.08
Cr10		Nominal	6.96	23.26	23.26	23.26	23.26
		EDS	6.38 ± 0.12	23.34 ± 0.25	24.18 ± 0.17	22.99 ± 0.15	23.11 ± 0.15
Cr15		Nominal	6.26	20.83	20.83	20.83	31.25
		EDS	5.87 ± 0.09	21.26 ± 0.24	20.08 ± 0.14	20.44 ± 0.13	32.35 ± 0.18

**Table 3:** Polarisation test results of the studied alloys in 3.5 wt.% NaCl solution.

Alloys	i_corr_, μA/cm^2^	E_pit_, mV	E_corr_, mV	E_pit_-E_corr_, mV
Cr0	0.798 (±0.157)	/	−294 (±12)	/
Cr3	0.145 (±0.037)	84 (±5)	−242 (±11)	326 (±16)
Cr5	0.141 (±0.042)	200 (±11)	−245 (±12)	445 (±23)
Cr10	0.129 (±0.036)	308 (±13)	−169 (±9)	477 (±22)
Cr15	0.071 (±0.014)	412 (±20)	−166 (±9)	578 (±29)

**Table 4:** The fitting parameter of EIS results for the studied alloys.

Alloys	Rs, Ω·cm^−2^	CPE_f_, μΩ^−1^·cm^2^·S^n^	n1	Rf, kΩ·cm^−2^	CPE_dl_, μΩ^−1^·cm^2^·S^n^	n2	Rct, kΩ·cm^−2^	Rp, kΩ·cm^−2^
Cr0	7.1 ± 0.09	36.4 ± 0.08	0.95 ± 0.002	7.6 ± 0.01	121.1 ± 0.038	1.00 ± 0.002	17.5 ± 0.91	25.1 ± 0.92
Cr3	7.9 ± 0.08	18.7 ± 0.05	1.00 ± 0.002	22.9 ± 0.03	29.3 ± 0.012	0.90 ± 0.002	240.5 ± 12.07	263.4 ± 12.10
Cr5	7.3 ± 0.12	18.4 ± 0.04	1.00 ± 0.003	27.4 ± 0.02	22.6 ± 0.009	0.91 ± 0.002	285.7 ± 14.91	313.1 ± 14.93
Cr10	7.5 ± 0.13	15.3 ± 0.03	1.00 ± 0.002	28.3 ± 0.03	14.3 ± 0.004	0.96 ± 0.003	496.5 ± 23.65	524.8 ± 23.68
Cr15	8.1 ± 0.15	16.1 ± 0.04	1.00 ± 0.002	36.5 ± 0.03	14.2 ± 0.005	0.95 ± 0.002	628.5 ± 25.32	665.0 ± 25.35

**Table 5:** The fitting parameter of EIS results for the studied alloys after immersion test.

Alloys	Time	Rs, Ω·cm^−2^	CPE_f_, μΩ^−1^·cm^2^·S^n^	n1	Rf, kΩ·cm^−2^	CPE_dl_, μΩ^−1^·cm^2^·S^n^	n2	Rct, kΩ·cm^−2^	Rp, kΩ·cm^−2^
Cr0	0 d	8.6 ± 0.15	24.5 ± 0.06	1.00 ± 0.003	15.6 ± 0.02	104.9 ± 0.035	1.00 ± 0.003	10.2 ± 0.51	25.8 ± 0.53
	1 d	8.2 ± 0.14	26.3 ± 0.07	0.96 ± 0.002	0.01 ± 0.00	16.8 ± 0.005	0.94 ± 0.003	17.5 ± 0.92	17.51 ± 0.92
	3 d	8.2 ± 0.16	16.3 ± 0.04	1.00 ± 0.003	0.01 ± 0.00	17.7 ± 0.006	0.94 ± 0.002	24.9 ± 1.37	24.91 ± 1.37
	5 d	8.0 ± 0.15	18.0 ± 0.05	0.98 ± 0.002	0.01 ± 0.00	25.8 ± 0.012	0.90 ± 0.002	31.0 ± 1.55	31.01 ± 1.55
	7 d	7.5 ± 0.13	24.6 ± 0.07	0.96 ± 0.002	0.001 ± 0.00	15.5 ± 0.006	0.94 ± 0.002	28.6 ± 1.33	28.60 ± 1.33
Cr3	0 d	7.9 ± 0.14	18.6 ± 0.04	1.00 ± 0.002	22.6 ± 0.03	29.3 ± 0.013	0.90 ± 0.002	240.8 ± 12.07	263.4 ± 12.10
	1 d	8.0 ± 0.15	14.6 ± 0.03	1.00 ± 0.003	49.5 ± 0.05	16.3 ± 0.005	0.94 ± 0.003	1252.0 ± 67.56	1301.5 ± 67.61
	3 d	7.7 ± 0.08	15.7 ± 0.03	1.00 ± 0.002	44.2 ± 0.04	10.8 ± 0.004	0.92 ± 0.002	1879.0 ± 98.56	1923.2 ± 98.60
	5 d	6.2 ± 0.06	24.1 ± 0.08	0.95 ± 0.003	738.2 ± 0.81	2.1 ± 0.000	0.60 ± 0.001	6622.0 ± 313.6	7360.2 ± 314.41
	7 d	6.2 ± 0.06	16.1 ± 0.04	1.00 ± 0.003	49.5 ± 0.05	11.5 ± 0.004	0.93 ± 0.003	2758.0 ± 147.1	2807.5 ± 147.15
Cr5	0 d	7.5 ± 0.08	15.7 ± 0.04	1.00 ± 0.002	35.7 ± 0.03	18.9 ± 0.008	1.00 ± 0.003	331.4 ± 17.56	367.1 ± 17.59
	1 d	11.8 ± 0.14	24.0 ± 0.07	0.95 ± 0.002	385.1 ± 0.42	5.1 ± 0.002	0.76 ± 0.001	1400.0 ± 81.22	1785.1 ± 81.64
	3 d	6.2 ± 0.08	11.2 ± 0.03	1.00 ± 0.003	59.0 ± 0.06	13.1 ± 0.005	1.00 ± 0.003	1594.0 ± 87.35	1653.0 ± 87.41
	5 d	5.4 ± 0.07	19.6 ± 0.06	0.96 ± 0.002	34.7 ± 0.04	2.4 ± 0.000	0.68 ± 0.001	5662.0 ± 280.2	5696.7 ± 280.24
	7 d	5.2 ± 0.07	20.3 ± 0.05	0.96 ± 0.002	383.1 ± 0.36	1.7 ± 0.000	0.49 ± 0.001	17,000 ± 755.6	17,383.1 ± 755.96
Cr10	0 d	7.9 ± 0.09	13.4 ± 0.04	1.00 ± 0.003	39.1 ± 0.05	15.1 ± 0.005	1.00 ± 0.003	461.5 ± 26.97	500.6 ± 27.02
	1 d	25.8 ± 0.28	14.5 ± 0.04	1.00 ± 0.002	62.6 ± 0.06	6.4 ± 0.003	0.92 ± 0.002	3061.0 ± 161.6	3123.6 ± 162.2
	3 d	7.2 ± 0.07	9.9 ± 0.02	1.00 ± 0.003	70.2 ± 0.08	6.6 ± 0.003	1.00 ± 0.003	3015.0 ± 170.4	3085.2 ± 171.2
	5 d	7.2 ± 0.07	15.8 ± 0.04	0.97 ± 0.003	138.9 ± 0.15	3.2 ± 0.000	0.87 ± 0.002	4745.0 ± 245.0	4883.9 ± 245.15
	7 d	6.6 ± 0.06	11.0 ± 0.03	1.00 ± 0.002	86.6 ± 0.09	7.6 ± 0.002	1.00 ± 0.003	5775.0 ± 301.4	5861.6 ± 301.49
Cr15	0 d	7.7 ± 0.08	12.5 ± 0.05	1.00 ± 0.003	43.0 ± 0.04	12.0 ± 0.004	0.99 ± 0.003	610.4 ± 33.92	653.4 ± 33.96
	1 d	6.9 ± 0.06	20.4 ± 0.07	0.95 ± 0.002	1388 ± 1.72	7.4 ± 0.004	0.93 ± 0.002	2107.0 ± 105.9	3495.0 ± 107.62
	3 d	7.4 ± 0.08	10.9 ± 0.03	1.00 ± 0.002	91.3 ± 0.12	5.5 ± 0.001	1.00 ± 0.003	2782.0 ± 127.1	2873.3 ± 127.22
	5 d	6.3 ± 0.05	15.4 ± 0.04	0.98 ± 0.003	90.1 ± 0.11	4.3 ± 0.002	0.85 ± 0.002	4129.0 ± 223.2	4219.1 ± 223.31
	7 d	6.5 ± 0.06	7.6 ± 0.03	1.00 ± 0.003	0.01 ± 0.00	11.8 ± 0.004	0.91 ± 0.002	4096.0 ± 211.9	4096.01 ± 211.9

## Data Availability

The raw data required to reproduce these findings can be obtained by contacting the corresponding author.
